# Burial of Soldiers

**Published:** 1862-05

**Authors:** 


BURIAL OF SOLDIERS.—By a recent regulation of the army it is made the duty of commanding generals to lay off lots of ground in some suitable spot near every battle-field, and cause the remains of those killed to be interred, with head-boards to the graves, bearing the numbers, and, where practicable, the names of the persons buried. A register of each burial-place, with these numbers and names, is to be kept. Friends may thus be enabled to know the resting-place of their dead. Apropos to the same subject, the Military Committee of the House have matured a bill for a National Cemetery in the District of Columbia, which will soon be reported to the House.

